# The impact of training and education programs for healthcare professionals on video- and text-based consultations in ensuring healthcare quality: a scoping review

**DOI:** 10.3389/fdgth.2026.1861018

**Published:** 2026-07-15

**Authors:** Md Shafiqur Rahman Jabin, Nussrat Bi, Shiji Thomas, Aneekah Ashfaq, Evalill Nilsson

**Affiliations:** 1 Department of Medicine and Optometry, Linnaeus University, Kalmar, Sweden; 2 Faculty of Health Studies, University of Bradford, United Kingdom; 3 School of Health and Society, University of Salford, Manchester, United Kingdom; 4 Faculty of Management, Law and Social Sciences, University of Bradford, Bradford, United Kingdom

**Keywords:** clinical competencies, curriculum design, digital transformation, health services delivery, implementation science, interprofessional education, organizational readiness, professional development

## Abstract

**Background:**

The rapid expansion of video- and text-based consultations has transformed healthcare delivery, particularly during and after the COVID-19 pandemic. As virtual care becomes embedded in routine practice, healthcare professionals require appropriate training to deliver safe and effective digital healthcare.

**Objective:**

This scoping review aimed to map and synthesize evidence on training and education programs designed to prepare healthcare professionals for video- and text-based consultations and to examine reported outcomes, facilitators, and barriers.

**Methods:**

This review followed Joanna Briggs Institute methodology and was reported in accordance with PRISMA-ScR and PRISMA-S guidelines. Multiple bibliographic databases and grey literature sources were searched for studies published between January 1, 2003, and December 24, 2024. Eligible studies included those involving healthcare professionals receiving training in video consultations or text-based communication. Two reviewers independently conducted study selection and data extraction. Findings were synthesized descriptively.

**Results:**

Twenty studies were included. Training approaches varied considerably and included workshops, webinars, online modules, simulations, telehealth Objective Structured Clinical Examinations, and hybrid programs. Most studies reported improvements in confidence, perceived competence, and readiness to provide virtual care. However, outcomes were predominantly based on short-term self-reported measures, with limited evidence relating to long-term skill retention, patient outcomes, or organizational impact. Most included studies focused on video-based consultations, while evidence relating specifically to text-based consultation training was limited. Common challenges included virtual physical examinations, technological barriers, and maintaining patient engagement.

**Conclusions:**

Telehealth training programs are increasingly used to support healthcare professionals in delivering video- and text-based consultations. The available evidence is concentrated on learner-focused outcomes, whereas patient-level, organizational, and long-term outcomes remain underexplored. To our knowledge, this is the first scoping review to synthesize training approaches across both video- and text-based consultation modalities. Future research should prioritize longitudinal evaluation, patient and organizational outcomes, and modality-specific competency development to support sustainable digital healthcare delivery.

## Introduction

Telehealth is broadly defined as the use of communication technologies to deliver healthcare at a distance (1). Advances in internet-based mobile technologies in the early 2000s expanded access to remote communication, making telehealth more feasible and scalable across settings (2, 3).

The COVID-19 pandemic accelerated the integration of digital technologies into routine healthcare delivery. In response to social distancing and travel restrictions, healthcare systems rapidly expanded video consultations and asynchronous communication tools, such as email, chat, and web portals, to maintain continuity of care (4, 5). Although telehealth services were established prior to the COVID-19 pandemic, the pandemic substantially accelerated their adoption and integration into routine healthcare delivery across a wide range of clinical settings (6).

Video consultations are used across diverse clinical contexts, including emergency and acute care (7, 8). Telehealth has also enabled triage of respiratory symptoms, post-discharge monitoring, chronic disease management, and advanced care planning, reducing unnecessary in-person visits and exposure risks (9). Remote patient monitoring technologies further support continuity of care and may contribute to reduced hospital readmissions (10). In long-term and residential care settings, virtual bedside consultations can limit unnecessary transfers while maintaining timely clinical assessment (8, 11). Telehealth has additionally expanded access to mental health services, behavioral interventions, and nutrition counseling (12–15).

The increasing use of digital technologies in healthcare has highlighted the importance of developing healthcare professionals' competencies to effectively engage with digital systems, support patient participation, and integrate technology into clinical practice (16–24). Virtual care can reduce geographical barriers and enhance accessibility for rural and underserved populations (17, 25). However, digital inequities, particularly among marginalized and Indigenous communities, continue to affect access to and engagement with digital health services (26, 27). Beyond patient access, video platforms also facilitate interdisciplinary collaboration, enabling shared decision-making and coordinated care across settings (28, 29).

As healthcare systems undergo digital transformation, clinicians increasingly work within technology-mediated environments (30–32). Effective implementation of digital health tools requires not only technical proficiency but also awareness of ethical, legal, and operational implications, including data privacy, patient safety, and system-related risks (33–36). Prior research has emphasized the need for improved reporting systems and classification frameworks to address health information technology–related incidents (32). These developments highlight the importance of structured education and ongoing professional development in digital competencies (14, 37, 38). Competency frameworks increasingly emphasize skills in conducting video- and text-based consultations, integrating digital tools into workflows, and evaluating their impact on quality of care and system performance (30, 31, 33–36, 38).

For the purposes of this review, video-based consultations are defined as synchronous interactions involving real-time audiovisual communication between healthcare professionals and patients. Text-based consultations are written digital communications, including secure messaging, email, chat functions, and patient portals, which may occur synchronously or asynchronously depending on the platform used. Traditional telephone consultations were outside the scope of this review because the focus was on digital communication modalities that rely primarily on video- or text-based technologies and require specific digital communication competencies.

The widespread adoption of video- and text-based consultations has transformed healthcare delivery. Despite this rapid expansion, variability persists in how healthcare professionals are trained to deliver safe, effective, and high-quality virtual care (6, 39). Existing studies examining telehealth education are dispersed across disciplines and differ substantially in design, delivery modes, and outcome measures (19, 40).

To date, no scoping review has comprehensively mapped the range of training and education programs specifically designed to prepare healthcare professionals for video- and text-based clinical encounters (6, 41–43). The protocol for this review was previously published in JMIR Research Protocols (6), and the present manuscript reports the final results and synthesis.

Video-based and text-based consultations represent two important but distinct modalities of digital healthcare delivery. Video consultations typically involve synchronous, real-time interactions that enable visual assessment, verbal communication, and rapport building, whereas text-based consultations, including secure messaging, email, chat functions, and patient portals, are generally asynchronous and rely primarily on written communication. Despite these differences, both modalities require healthcare professionals to develop digital communication competencies, adapt clinical workflows, address privacy and confidentiality considerations, and maintain healthcare quality in virtual settings (5, 11, 17, 39). Examining these modalities together provides a broader understanding of how training and education programs prepare healthcare professionals for contemporary digital healthcare practice.

Previous reviews have examined telehealth workforce preparedness and competency development among healthcare professionals, including the scoping review by Browne et al. on preparing the workforce for telehealth practice (53, 78). However, these reviews have generally focused on broader telehealth capability development rather than specifically examining training and education programs designed to support video- and text-based consultations. Furthermore, limited attention has been given to synthesizing reported outcomes, facilitators, barriers, and evidence gaps across different consultation modalities and healthcare professions. The present review addresses this gap by providing a focused synthesis of training approaches for video- and text-based consultations across diverse healthcare contexts.

This review synthesizes available evidence to identify training approaches, examine reported facilitators and barriers, and analyze the perceived impact of these programs from the perspectives of healthcare professionals, patients, and healthcare organizations. By consolidating this evidence, the review aims to inform curriculum development, policy planning, and future research in digital health education.

This scoping review mapped and synthesized the characteristics of training programs designed to prepare healthcare professionals to conduct video- and text-based consultations and examined the outcomes reported in the literature. The review examined how these programs were implemented and assessed their reported impact.

The review addressed the following questions:

- What training and education programs are used to train healthcare professionals in conducting video- and text-based meetings (emails, chats, web portals)?
- What are the facilitators and barriers in conducting video- and text-based meetings?
- What are the impacts of such training and education programs from the perspectives of patients, healthcare professionals, and the healthcare organization?

## Methods

### Protocol and registration

This scoping review was conducted in accordance with the Joanna Briggs Institute (JBI) methodology for scoping reviews (43–48). The protocol for this review was prospectively developed and published in JMIR Research Protocols (6). The present manuscript reports the final results of that review.

The review was conducted largely as described in the published protocol (6). Minor refinements were made to enhance the transparency and comprehensiveness of reporting. These refinements did not alter the review questions, eligibility criteria, or overall methodological framework.

This review is reported in accordance with the PRISMA-ScR (Preferred Reporting Items for Systematic Reviews and Meta-Analyses Extension for Scoping Reviews) checklist (49) (see Supplementary Multimedia Appendix 1), and the search strategy is reported in accordance with PRISMA-S recommendations to strengthen transparency and reproducibility.

### Eligibility criteria

The review followed the Population–Concept–Context (PCC) framework recommended by JBI (43–46) outcome measureStudies focusing exclusively on traditional telephone consultations were excluded because the review specifically examined training and education programs relating to video- and text-based digital consultation modalities.

#### Population

Eligible studies included healthcare professionals (including students and qualified practitioners) who received training or education in conducting video- or text-based consultations. Healthcare professionals included physicians, nurses, allied health professionals, behavioral health providers, trainees, and interdisciplinary teams.

#### Concept

The core concept was educational or training interventions designed to prepare healthcare professionals to conduct video consultations or text-based communication (e.g., email, chat functions, web portals) in clinical settings. Studies evaluating implementation, perceived competence, barriers, facilitators, readiness, or outcomes of such training were included.

#### Context

Studies conducted in healthcare settings, including hospitals, primary care settings, outpatient clinics, long-term care facilities, academic institutions delivering clinical education, and community healthcare settings, were eligible.

### Types of evidence sources

Consistent with scoping review methodology, a broad range of study designs was included, encompassing randomized and non-randomized controlled trials, quasi-experimental studies, cohort and cross-sectional studies, qualitative and mixed-methods studies, as well as case studies and action research.

A scoping review methodology was selected because the objective was to map the extent, characteristics, and nature of the available evidence relating to telehealth training and education programs rather than evaluate intervention effectiveness. Given the anticipated heterogeneity of study designs, populations, training approaches, and outcome measures, a scoping review was considered the most appropriate approach in accordance with JBI guidance.

#### Additional criteria

Only studies published in English were included. Searches were limited to publications from January 1, 2003, onward to reflect the emergence of modern internet-enabled telehealth systems and to ensure consistency across databases. The year 2003 was selected as the start date to capture the early expansion of telehealth and digitally mediated consultation technologies within healthcare practice and professional education while maintaining a sufficiently broad timeframe for evidence mapping.

#### Information sources

A comprehensive search strategy was developed in consultation with academic librarians. The following bibliographic databases were searched: PubMed (MEDLINE via PubMed), CINAHL (via EBSCOhost), PsycINFO (via EBSCOhost), and APA PsychArticles (via EBSCOhost). Grey literature sources included OCLC WorldCat and ProQuest Dissertations and Theses.

Citation tracking and manual screening of reference lists of included studies were conducted to identify additional relevant publications. The final search was conducted on December 24, 2024.

### Search strategy

The search strategy was developed iteratively in line with JBI guidance (43–46) and informed by the JBI Manual for Evidence Synthesis (50). Controlled vocabulary (e.g., MeSH terms in PubMed and subject headings in CINAHL and PsycINFO) and free-text terms were combined using Boolean operators (AND, OR). Truncation symbols, phrase searching, and database-specific field modifiers (e.g., title and abstract fields) were applied as appropriate. Synonyms and related terms were incorporated for key concepts, including telehealth, telemedicine, video consultation, virtual care, digital communication, text-based communication (e.g., email, chat, and web portals), as well as training, education, and competency. The strategy was refined in consultation with academic librarians to maximize sensitivity while maintaining the feasibility of screening. Complete database-specific search strategies, including controlled vocabulary, Boolean strings, field tags, truncation symbols, applied limits, and numbers of records retrieved, are provided in Supplementary Multimedia Appendix 2 in accordance with PRISMA-S guidance (49).

### Selection of sources of evidence

All identified records were exported to EndNote 20 (Clarivate Analytics), where initial duplicate removal was performed. Records were subsequently uploaded to Covidence for screening. Duplicate removal occurred in two stages: automatic removal in Covidence (*n* = 1,014), followed by manual verification (*n* = 1).

Two independent reviewers screened titles and abstracts against the eligibility criteria. Potentially relevant studies were retrieved for full-text assessment. Full texts were independently assessed by at least two reviewers. Disagreements were resolved through discussion and, where necessary, consultation with an additional reviewer. Reasons for exclusion at the full-text stage were documented and are presented in the PRISMA 2020 flow diagram (48).

### Data charting process

Data extraction (charting) was conducted using a structured data extraction form developed by the research team in accordance with JBI guidance (50).

The charting form was piloted on a subset of included studies and refined to improve clarity and consistency. Data were independently extracted by at least two reviewers to enhance reliability. The data charting process was iterative, allowing refinement of categories during full-text review, consistent with scoping review methodology.

#### Data items

The following variables were extracted from each included study: author(s), year, and country; study design; setting and context; participant characteristics; type and format of the training intervention; duration and delivery mode; technologies used; outcomes measured; reported facilitators and barriers; reported impacts at the levels of healthcare professionals, patients, and organizations; and key findings with author recommendations.

These data items were selected to align directly with the review questions and to facilitate descriptive mapping of training interventions and their reported impacts.

#### Evidence stratification

To enhance the internal validity and interpretability of the findings, the included studies were stratified by study design and methodological robustness. Studies were grouped into quantitative, qualitative, mixed-methods, and other designs (e.g., case studies and action research). This stratification enabled the contextual interpretation of findings, particularly regarding the strength and limitations of the evidence base. While formal critical appraisal was not conducted in accordance with JBI guidance for scoping reviews (43–46), this approach allowed for a more structured and transparent synthesis of heterogeneous evidence.

### Critical appraisal of individual sources

No formal methodological quality appraisal or risk-of-bias assessment was conducted. The purpose of this review was to map and synthesize the range and characteristics of available evidence rather than to evaluate intervention effectiveness.

### Synthesis of results

The extracted data were synthesized descriptively using a convergent-integrated approach. Quantitative, qualitative, and mixed-method findings were integrated to identify patterns across training approaches, delivery modalities, duration and structure, reported outcomes, facilitators and barriers, and perceived impacts on healthcare professionals, patients, and organizations.

The synthesis focused on mapping the breadth and characteristics of telehealth training programs rather than conducting statistical meta-analysis. Findings were organized thematically in alignment with the review questions. This approach aligns with JBI guidance on the presentation and analysis of results in scoping reviews, emphasizing structured data charting, evidence mapping, and identification of knowledge gaps (50).

A formal evidence gap map was considered during the synthesis process; however, it was not developed due to substantial heterogeneity in study designs, intervention types, and outcome measures, and the limited number of studies within comparable categories, which limited the feasibility of constructing a meaningful and interpretable gap map.

## Results

### Search results and study selection

The database and grey literature search identified 3,976 records, comprising 3,420 records from bibliographic databases and 556 from grey literature and other sources. After removal of duplicates (*n* = 995), 2,981 records were screened at the title and abstract level, reflecting a broad and sensitive search strategy. Of these, 2,935 were excluded as not meeting the eligibility criteria.

**Figure 1:**
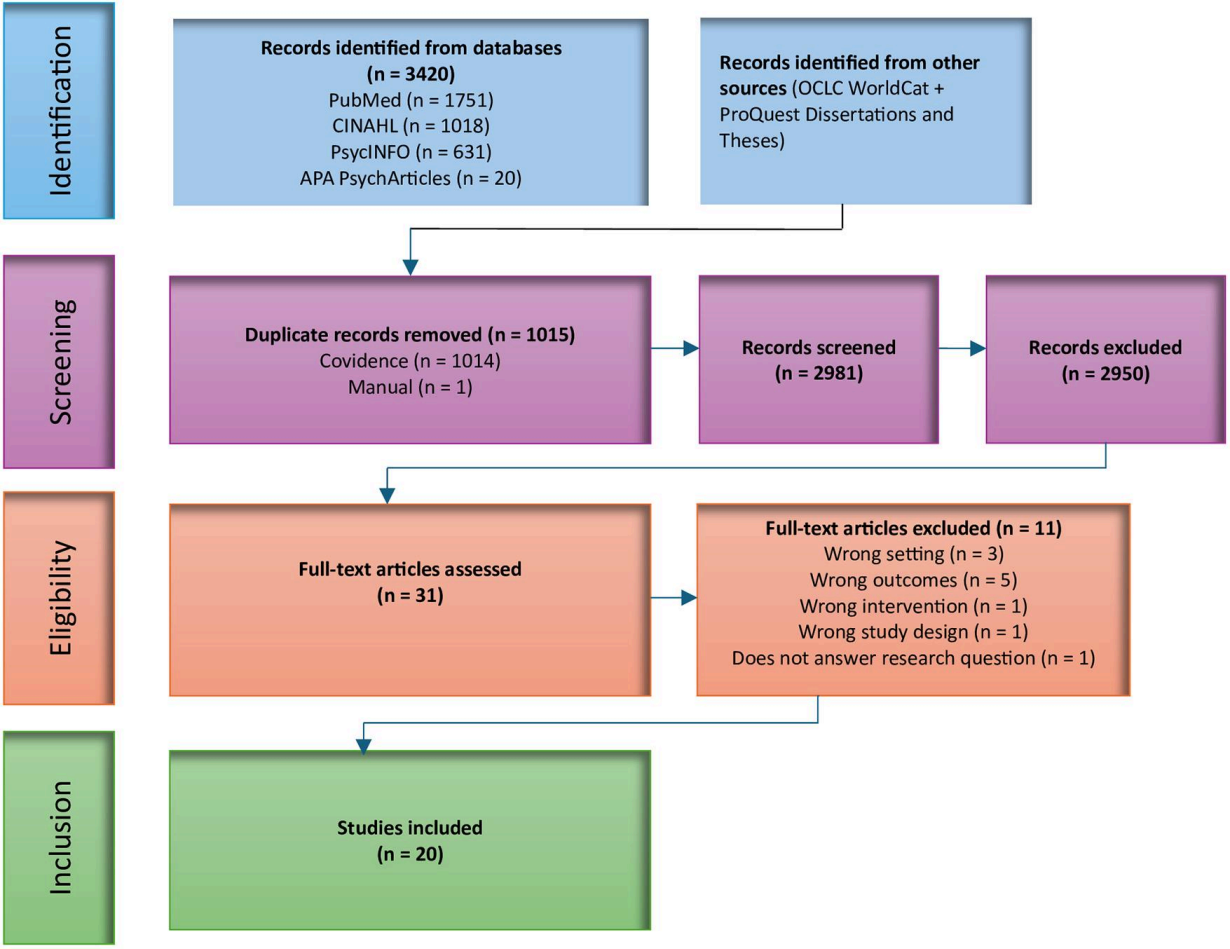
PRISMA 2020 flow diagram illustrating the study selection process for the scoping review.

Thirty-one full-text articles were assessed for eligibility. Eleven studies were excluded because they did not meet the inclusion criteria (e.g., population, intervention, context, or study design). Twenty studies were included in the final synthesis. The study selection process is presented in the PRISMA 2020 flow diagram (Figure 1) in accordance with updated reporting guidance (48).

### Characteristics of included studies

The 20 included studies comprised 10 quantitative, 6 mixed-methods, 2 qualitative, 1 case, and 1 action-research study. Quantitative and mixed-methods studies primarily contributed evidence on measurable outcomes such as confidence, competence, and training effectiveness, whereas qualitative and descriptive studies provided contextual insights into implementation processes, perceived barriers, and experiential aspects of telehealth training.

Studies were conducted across seven countries: the United States, South Africa, the United Kingdom, Nigeria, the Netherlands, Switzerland, and Australia (24, 51–69). Most studies were conducted in academic or clinical training environments, including medical schools, nursing schools, rural healthcare settings, and hospital-based programs. Publication increased substantially after 2020, reflecting accelerated telehealth adoption during the COVID-19 pandemic (24, 51–62, 64–69). Participants included medical students (24, 51, 53), nursing and advanced practice nursing students (55, 58, 59, 67), behavioral health providers (52, 64), psychiatrists and psychotherapists (56, 60), rural doctors (57), allied health professionals (54, 62), and interdisciplinary trainees (58).

Of the 20 included studies, 17 primarily focused on video-based consultations or telehealth encounters delivered through synchronous communication technologies, while 3 studies addressed broader telehealth, eHealth, or mobile health communication approaches. No included study focused exclusively on training healthcare professionals for text-based consultation modalities such as secure messaging, email consultations, web portals, or chat-based communication. Consequently, the available evidence is heavily weighted toward video-based telehealth training.

To enhance the presentation of the evidence, the extracted data were systematically charted and organized across key dimensions, including study design, participant characteristics, type of training intervention, delivery mode, and reported outcomes. This structured data charting enabled comparison across studies and supported identification of patterns, trends, and evidence gaps within the existing literature.

The characteristics of the included studies, including study design, setting, participants, intervention characteristics, outcomes, key findings, and recommendations, are summarized in Table 1. A summary evidence-mapping table presenting profession, consultation modality, training format, duration, and outcome domains across included studies is provided in Table 2.

**Table 1:** Characteristics of included studies examining training and education programs for healthcare professionals conducting video- and text-based consultations.

No.	Study/Country/Year	Type of study	Setting	Participants	Intervention(s)	Outcomes measured/Phenomena of interest	Claimed findings	Further suggestions
1.	Gifford et al. (United States) (2012) (16)	Quantitative	Behavioural HC services	21 behavioral health providers completed the training	Three-day face-to-face behavioral TH training program. Participants watched two videos, role-played clinical scenarios, and had group discussions.	Behavioral TH competency improvement	Improvements in behavioral TH competencies	Develop best practices and enhance assessment tools for TH
2.	Scott and Mars (South Africa and Canada) (2014) (63)	Action research	Global eHealth landscape	HC professionals, support staff, policy makers, administrators, the general population, and students	Development and implementation of structured e-health training	Effective strategies and technologies to enhance e-Health training and capacity building	A comprehensive, structured approach to e-Health training is essential	Develop a generic TH curriculum
3.	Gunner et al. (United Kingdom) (2021) (53)	Qualitative	Highlands Medical Education Centre	40 final year MS	A teaching module for video consultation skills Two-hour face-to-face teaching sessions	Effectiveness of the teaching module on video consultation skills	Training improved students’ confidence and abilities significantly	Improve the video consultation skills module
4.	Powell et al. (United States) (2022) (66)	Mixed methods	204 NH	21 NH administrators and clinicians		Identifying facilitators and barriers to TH implementation in NH	Training and technology are key facilitators for those who have improved their TH practices	Improve staff training, optimizing workflows with checklists to enhance TH implementation, better data interoperability, and resident feedback
5.	Nearing et al. (United States) (2020) (61)	Mixed methods	GRECC within the Veterans Health Administration	Older Rural Adults, altogether 1,000 participants (300 pre-registered plus 700 who attended)	Webinar. Presentation slides, recorded webinar, and chat transcript stored on SharePoint	Enhancing TH training for geriatric care amidst the challenges posed by COVID-19,	Identified significant training gaps among trainees. GRECC Connect rapidly developed educational resources and webinars.	Follow-up surveys in six months to evaluate the effectiveness of the training
6.	Iliyasu et al. (Nigeria) (2024) (65)	Mixed methods	Hospitals and clinics	246 physicians	Questionnaires and interviews to assess TM practices and challenges	Assessing physicians’ knowledge, attitudes, adoption factors, and challenges related to the use of TM during the COVID-19 pandemic	TM use increased significantly during the pandemic, influenced by experience and training	Enhance technology and clinician training for TM
7.	Mishkin et al. (United States) (2022) (60)	Quantitative	Consultation-Liaison Psychiatry settings	333 clinicians (psychiatrists and psychotherapists)	The use of TP	Overall Satisfaction, completeness, and patient Satisfaction	85.9% of respondents reported overall satisfaction with TP	Enhance TP training
8.	Browne et al. (United States) (2022) (58)	Qualitative	2 rural health clinics	19 nurse practitioner students, 14 social work students, 10 pharmacy students, and one public health student	An interprofessional TH course	Student TH's competence and patient benefits	Increased students’ confidence and skills, and improved patient mental health and empowerment	Include patient interviews and long-term outcome assessments
9.	Nohelty et al. (United States) (2021) (64)	Case study	Clinics	Two BT, two patients	To assess the effectiveness and quality of TH's direct therapy sessions	BT skills, patient engagement, caregiver support, BIP fidelity, skill acquisition, and data collection	Identified gaps in BT skills during TTTIM sessions, leading to targeted professional goals and feedback	Refine the TTTIM and develop variations for different patient needs
10.	Samuels et al. (United States) (2022) (68)	Quantitative	Children's Medical Centre	33 pediatric interns	Self-directed, five-unit online curriculum	Privacy assurance, rapport establishment, demonstration of empathy, and partnership-building	Interns who completed the TM curriculum improved their communication scores	Develop a longitudinal learning platform to enhance TM skills
11.	Bajra et al. (United States) (2023) (51)	Mixed methods	Stanford School of Medicine	133 third- and fourth-year MS	Interactive TH Workshop, TH TeleOSCEs, enhance MS TH skills and self-efficacy, and patient encounters	Self-efficacy in TH, Perceptions of Quality of Care, Performance in TeleOSCEs, Student Feedback, and Reflections	TH competencies can be successfully taught and assessed in medical education	Improve training on TH limitations
12.	Boardman et al. (United States) (2021) (24)	Mixed methods	Simulation Centre for the Health Sciences	23 participants from the Internal Medicine residency program	A training guide on video-based communication skills and YouTube tutorials on virtual physical examination were provided	Communication Skills, Patient Activation and Satisfaction, Telemedicine Skills	Residents excelled in nonverbal communication but struggled with virtual exams and patient education	More virtual practice opportunities and integrating TM training into the residency curriculum
13.	Holdsworth et al. (United Kingdom) (2021) (54)	Quantitative	HC facilities	2,320-live webinars, 3,884-viewed the recordings	To facilitate the rapid upskilling of AHPs to ensure continuity of care while adhering to safety protocols	Knowledge and Confidence Levels, Utilization of Video Conferencing, Consultation Statistics	The webinars were effective in enhancing AHPs’ readiness to implement VC in their practice	Enhance the integration of VC into HC
14.	Rusu et al. (Netherlands) (2023) (56)	Quantitative	5 specialized CAP centres	1,065 clinicians	Transition from in-person treatment to telepsychiatry for child and adolescent psychiatric care	Clinicians’ Impressions of TP, Training and Use of TP, Self-Assessment of Competence	Clinicians with prior training and experience in TP had positive impressions compared to those without	Integrate training with clinical practice in TP to better support clinicians
15.	Leone et al. (United Kingdom) (2022) (62)	Mixed method	Hospital Outpatient/Inpatient, Clinics, Community Health Services, Primary Care, Domiciliary Services	539 clinicians, 119 as managers	Facilitate transition to TH services, ensuring that AHPs were well-equipped to provide effective remote care	Training and Skills, TH Guidelines Implementation, Perceived Barriers	The progress made in adopting TH practices and the critical areas needing improvement	Create standardized TH guidelines and improve training for AHPs, especially for vulnerable populations
16.	Jacob et al. (United Kingdom and Switzerland) (2020) (69)	Review- Qualitative	Hospitals, clinics, Long-term care facilities, and health centres	Physicians, Nurses, MS, Patients, Caregivers	Understanding clinicians’ adoption of MHealth tools	Clinicians’ adoption of MHealth	Existing frameworks for mHealth adoption need expansion or combination to address the complex factors influencing clinicians	Include non-English research and grey literature, exploring more theoretical frameworks
17.	Taylor et al. (Australia) (2024) (57)	Mixed methods	Rural and remote healthcare settings	11 doctors	TCSP Online workshops	Confidence and Competence, Training Effectiveness, Technology Awareness	TCSP successfully equipped rural doctors with the necessary skills and confidence to conduct effective remote consultations	Increase participant numbers and ongoing technical support
18.	List et al. (United States) (2019) (67)	Quantitative (Quality improvement survey)	A school of nursing in a private university	32 NS	Integrating TH learning outcomes into an FNP program using a hybrid approach face-to-face in campus classes and online classes	Student confidence in TH's knowledge	FNP confidence in TH knowledge significantly increased after the intervention	Explore integrating TH learning outcomes into clinical practicum courses
19.	Love and Carrington (United States) (2021) (55)	Mixed methods	University of Florida, College of Nursing	83 DNPs,	Equip DNP with the skills and confidence to provide TH services effectively by giving online access to reading materials, short videos, simulated telehealth visits, and multiple-choice questions	Student Preparedness, Patient Satisfaction, Technical Proficiency	NS valued telehealth but struggled with tech; patients enjoyed it but missed in-person contact.	Enhance training, address tech issues, and create a framework for diverse patients
20.	Chike-Harris et al. (United States) (2022) (59)	Quantitative	A nursing school	59 APRN	Self-paced online module on TH professionalism. Training method: video-recorded lectures, and face-to-face discussions	knowledge gains in TH professionalism and therapeutic communication	APRNs improved their knowledge of TH professionalism and therapeutic communication, increased confidence in managing depression in pregnancy	More experiential TH activities in the APRN curriculum

**Table 2:** Evidence map of included studies according to profession, consultation modality, training format, duration, and outcome domains.

No.	Study	Profession	Consultation modality	Training format	Duration	Outcome domain(s)
1	Gifford et al. (2012) (16)	Behavioral health providers	Video	Face-to-face workshop, role-play, discussion	3 days	Learner, clinical skills
2	Scott and Mars (2014) (63)	Healthcare professionals, administrators, students, policymakers	Both (Video/Text)	Structured eHealth training	NR	Organizational
3	Gunner et al. (2021) (53)	Medical students	Video	Face-to-face teaching module with simulated consultations	2 h	Learner, clinical skills
4	Powell et al. (2022) (66)	Nursing home administrators and clinicians	Video	Telehealth implementation and workflow training	NR	Organizational
5	Nearing et al. (2020) (61)	Geriatric healthcare providers	Video	Webinar, recorded materials, chat-based learning	NR	Learner
6	Iliyasu et al. (2024) (65)	Physicians	Video	Telemedicine training and assessment	NR	Learner
7	Mishkin et al. (2022) (60)	Psychiatrists and psychotherapists	Video	Telepsychiatry training and implementation	NR	Learner, patient
8	Browne et al. (2022) (58)	Nursing, social work, pharmacy, and public health students	Video	Interprofessional telehealth course	14 weeks	Learner, patient
9	Nohelty et al. (2021) (64)	Behaviour technicians	Video	Telehealth supervision using TTTIM	NR	Clinical skills, patient
10	Samuels et al. (2022) (68)	Pediatric interns	Video	Self-directed online curriculum	Self-paced	Clinical skills
11	Bajra et al. (2023) (51)	Medical students	Video	Interactive workshop and TeleOSCEs	Clerkship-based	Learner, clinical skills
12	Boardman et al. (2021) (24)	Internal medicine residents	Video	Video communication training and virtual examination tutorials	NR	Clinical skills, patient
13	Holdsworth et al. (2021) (54)	Allied health professionals	Video	Live webinars and recorded sessions	NR	Learner
14	Rusu et al. (2023) (56)	Child and adolescent psychiatry clinicians	Video	Telepsychiatry transition training	NR	Learner
15	Leone et al. (2022) (62)	Allied health professionals and managers	Both (Video/Text)	Telehealth implementation training	NR	Organizational
16	Jacob et al. (2020) (69)	Physicians, nurses, medical students, patients, caregivers	Both (Video/Text/Mobile Health)	Frameworks for mHealth adoption and training	NR	Organizational
17	Taylor et al. (2024) (57)	Rural doctors	Video	Telehealth Clinical Skills Program (TCSP), online workshops	NR	Learner, clinical skills
18	List et al. (2019) (67)	Nursing students	Video	Hybrid curriculum (face-to-face and online)	Course-based	Learner
19	Love and Carrington (2021) (55)	Doctor of Nursing Practice students	Video	Online learning platform with simulations	NR	Learner, patient
20	Chike-Harris et al. (2022) (59)	Advanced practice registered nursing students	Video	Self-paced online module with lectures and discussion	Self-paced	Learner, clinical skills

NR, not reported; TeleOSCE, telehealth objective structured clinical examination; TTTIM, telehealth training and telehealth integration model.

Of the 20 included studies, 17 focused primarily on video-based consultations, whereas 3 addressed broader telehealth, eHealth, or mobile health approaches incorporating both video- and text-based communication modalities. No study focused exclusively on text-based consultation training. Evidence was concentrated on learner and clinical skills outcomes, while patient and organizational outcomes were reported less frequently.

### Types of training interventions

Training approaches varied widely in instructional design, duration, delivery mode, and evaluation strategy.

#### Face-to-face training

Early interventions primarily involved in-person training. Gifford et al. delivered a three-day behavioral telehealth training program focusing on ethics, cultural considerations, and practical use of video technology (52). Gunner et al. provided a structured 2-hour session for medical students incorporating simulated consultations and structured feedback (53).

#### Blended and hybrid approaches

Several studies adopted blended formats combining recorded lectures with face-to-face discussion (59) or hybrid classroom and teleconferencing participation (67). These approaches aimed to balance theoretical knowledge with experiential learning.

#### Online and scenario-based training

Fully online or scenario-based training became more prominent in later studies. Taylor et al. delivered structured scenario-based training for rural doctors (57), while Samuels et al. implemented a self-directed online telemedicine curriculum for pediatric interns (68). Love and Carrington developed an online learning platform with simulated telehealth visits and knowledge assessments (55).

#### Multi-modal and experiential programs

More comprehensive programs integrated coursework, simulation, supervised telehealth encounters, and reflective practice. Browne et al. implemented a 14-week interprofessional telehealth course (58), and Bajra et al. embedded telehealth workshops and teleOSCEs within a medical clerkship (51). Boardman et al. assessed baseline performance through video-based OSCEs before providing targeted training (24).

#### Webinars and rapid upskilling

Webinars were used to rapidly upskill clinicians during pandemic-related shifts to remote care (54, 61), often supplemented with recorded materials and interactive chat features.

Overall, interventions ranged from brief sessions lasting a few hours to longitudinal programs extending over several months.

### Impact of telehealth training

Ten studies formally evaluated the impact of training programs on healthcare professionals (24, 51–59, 68).

#### Healthcare professional perspectives

Across studies, training was consistently associated with improvements in perceived confidence, competence, and readiness to deliver virtual care; however, these outcomes primarily reflect self-reported measures rather than objective performance or long-term behavioral change (53, 54, 67). Medical students reported improved consent processes and virtual examination skills following structured workshops and teleOSCEs (51, 53). Behavioral health providers reported enhanced telehealth competencies after targeted training (52).

Rural doctors and child and adolescent psychiatry clinicians with prior telehealth training reported greater confidence and more positive impressions of virtual care compared to those without training (56, 57). Interprofessional training improved understanding of professional roles and collaborative practice in telehealth contexts (58). However, clinicians also reported ongoing challenges related to technical disruptions and limitations in conducting virtual physical examinations (51, 55, 57, 60).

Differences between video-based and text-based modalities were not consistently distinguished across the included studies. However, where reported, video-based consultations primarily required competencies related to visual communication, non-verbal cues, and remote physical assessment, whereas text-based communication emphasized clarity of written communication, asynchronous interaction, and documentation practices. This distinction highlights the need for modality-specific training approaches, although such differentiation was limited in the current evidence base.

#### Patient perspectives

Although patient outcomes were not systematically evaluated across studies, patients participating in simulation or pilot telehealth initiatives reported convenience and perceived benefits, particularly in mental health contexts (55, 58). Standardized patients identified areas for improvement, including technical optimization and patient education during virtual encounters (24).

#### Organizational perspectives

Healthcare organizations and educators reported improvements in trainee communication, privacy management, and professional conduct following telehealth training interventions (58, 68). Some studies emphasized the importance of workflow optimization and standardized guidelines to support telehealth implementation (62).

### Facilitators and barriers to telehealth use

Formal training, prior telehealth experience, and positive attitudes toward digital tools were associated with increased adoption (65, 66). Barriers included inadequate infrastructure, limited access to devices or reliable internet, workflow disruptions, time constraints, and difficulty engaging patients with cognitive impairments (65, 66).

Frameworks such as the Technology Acceptance Model and diffusion of innovation theory have been used to explain clinician adoption of mobile and digital health tools, emphasizing perceived usefulness, training availability, and technical support (69).

### Reported challenges and recommendations

Commonly reported challenges included conducting virtual physical examinations (51, 55, 57), managing technical difficulties (51, 55, 57, 58), and maintaining rapport in virtual settings (51). Resource limitations and lack of inter-agency coordination also affected telehealth implementation (52, 58).

Several studies recommended enhanced experiential learning opportunities (24, 51, 56), stronger academic–clinical partnerships (67), integration of supervision tools such as the Telehealth Therapy Treatment Integrity Measure (TTTIM) (64), and development of contingency systems to mitigate technical disruptions (37, 55, 70, 71).

### Integrated synthesis

In line with scoping review methodology, the synthesis moved beyond descriptive reporting to identify patterns in the distribution of evidence, key areas of concentration, and gaps in the literature. The analysis focused on how training interventions varied across modalities, duration, and contexts, and how these variations influenced reported outcomes.

A comparative analysis of training approaches across the included studies reveals several consistent patterns. Online and webinar-based training formats were commonly used for rapid scalability and accessibility, particularly during the COVID-19 period, whereas face-to-face and simulation-based approaches were commonly reported in studies describing experiential learning and skills development. Short-duration interventions commonly reported introductory knowledge and communication-focused content, whereas longer or longitudinal programs more frequently reported practice-based learning and competency development components. Similarly, simulation-based training (e.g., teleOSCEs) emphasized structured skill acquisition and assessment, whereas real-world clinical training highlighted challenges related to workflow integration and patient interaction.

#### Heterogeneity of educational design

Across the included studies, training interventions varied substantially in structure, duration, and delivery modality. Programs ranged from brief workshops to longitudinal interprofessional curricula. Delivery formats included face-to-face sessions, fully online modules, hybrid learning environments, webinars, and simulation-based teleOSCEs (24, 51–59, 67, 68). This heterogeneity reflects rapid adaptation to the expansion of telehealth, particularly during the COVID-19 pandemic.

#### Predominance of confidence-based outcomes

Most studies evaluated short-term outcomes, primarily focusing on self-reported confidence, perceived competence, and readiness to deliver virtual care (51, 53–57, 67, 68). Objective performance measures were less common and often limited to simulated environments such as teleOSCEs (24, 51). Few studies assessed long-term behavioral change or patient-level outcomes.

In addition to telehealth-specific skills, several studies indirectly highlighted the importance of broader digital competencies, such as navigating digital systems, integrating technology into clinical workflows, and adapting to evolving digital infrastructures, suggesting that telehealth training may form part of a wider digital capability framework within healthcare practice.

#### Distribution of reported outcomes

The included studies reported outcomes across four broad domains: learner outcomes, clinical skills outcomes, patient outcomes, and organizational outcomes. Learner outcomes were the most frequently reported and included confidence, perceived competence, telehealth knowledge, and readiness to provide virtual care (51, 53–59, 67, 68). Clinical skills outcomes included communication, history-taking, virtual physical examination, professionalism, rapport-building, and patient education skills developed through simulation activities, teleOSCEs, and role-play exercises (24, 51, 53, 57, 68). Patient outcomes were reported less frequently and primarily focused on patient experiences, convenience, and perceived benefits of telehealth encounters (55, 58). Organizational outcomes were also limited and included workflow optimization, telehealth implementation, provider experiences, and integration of telehealth into practice (58, 62, 66, 68). Overall, the evidence was concentrated on learner and clinical skills outcomes, whereas comparatively little evidence examined patient-level or organizational impacts.

#### Limited organizational and system-level evaluation

While some studies considered workflow optimization and guideline availability (62, 66), most focused on individual learner outcomes. Evidence regarding organizational performance, system integration, and sustainable implementation remains limited.

#### Recurrent implementation challenges

Commonly reported barriers included technological disruptions, difficulty conducting virtual physical examinations, and challenges maintaining rapport (51, 55, 57). Facilitators consistently included prior experience, formal training, and positive attitudes toward telehealth (65, 66).

Overall, the findings provide a structured mapping of the available evidence, demonstrating that learner and clinical skills outcomes were the primary focus of existing studies, while patient-level, organizational, and long-term outcomes remain underrepresented.

## Discussion

### Principal findings

This scoping review mapped and synthesized evidence on training and education programs designed to prepare healthcare professionals to conduct video- and text-based consultations, focusing on identifying training approaches, facilitators, and barriers, and reporting impacts across professional, patient, and organizational levels. Overall, the findings indicate substantial variability in training design, delivery, and evaluation, with most studies reporting improvements in healthcare professionals' perceived confidence and readiness to deliver virtual care, while evidence on long-term, patient-level, and organizational outcomes remains limited.

A notable finding was the substantial imbalance between consultation modalities represented in the literature. Seventeen of the 20 included studies focused primarily on video-based consultations, whereas no study evaluated training exclusively designed for text-based consultations. As a result, the conclusions of this review are predominantly informed by evidence relating to synchronous video-based care. This limits the extent to which findings can be generalized to asynchronous communication modalities, such as secure messaging, email, patient portals, or chat-based consultations. The lack of modality-specific evidence highlights an important gap in the literature and underscores the need for future research on competencies, training approaches, and outcomes in text-based healthcare communication.

### Interpretation of findings

The findings suggest that telehealth training programs vary widely in their design, delivery, and evaluation, reflecting the evolving, context-dependent nature of digital healthcare education. These findings are broadly consistent with existing literature, which has reported similar variability in telehealth training approaches and limitations in evaluation methods.

The review identified a broad range of training formats, including traditional face-to-face sessions, online lectures, webinars, group discussions, video-based Objective Structured Clinical Examinations (OSCEs), simulated patient encounters, and access to recorded materials and guidelines. The absence of a standardized instructional model reflects both the adaptability of educational strategies and the evolving nature of telehealth practice (72, 73).

Most training programs were delivered through online platforms. This trend likely reflects both the urgency of telehealth integration during pandemic-related restrictions (4, 39) and the practical advantages of digital delivery formats. Online training may provide flexibility, scalability, and cost-efficiency (73, 74); however, comparative effectiveness across delivery modalities remains underexplored. This variability also extends to the content and competencies addressed within training programs.

Substantial variation was also observed in training content. Most programs focused on developing competencies in patient information gathering, virtual physical examination techniques, patient confidentiality, and the effective use of digital platforms. Only one study explicitly addressed the use of telehealth technologies to support interdisciplinary collaboration (55). Nonetheless, the competencies developed through telehealth training may extend to collaborative and team-based care contexts (40, 75).

Training duration ranged from brief sessions lasting a few hours to longitudinal interventions extending over several months. Similar variability in telehealth education design has been reported in previous reviews (73, 74). The mapped evidence indicates that telehealth training remains heterogeneous, with limited agreement regarding optimal curriculum structure or duration.

Beyond telehealth-specific skills, broader digital competencies, including electronic health record (EHR) navigation, awareness of artificial intelligence (AI), and understanding of interoperability, are increasingly recognized as essential for contemporary healthcare professionals (76). Incorporating such system-level competencies may enhance workflow integration and support the sustainability of digital care models. These differences in training design and content are reflected in the reported impacts on clinical practice and quality of care.

Several studies reported improvements in healthcare professionals' confidence, perceived competence, and readiness to deliver virtual care following participation in training interventions (51, 54, 56–58). Models and best practice examples for primary care teleconsultation training emphasize structured pedagogical design and practical application exercises (77). The frequent emphasis on improvements in confidence may reflect the immediate and subjective nature of training evaluations rather than deeper changes in clinical practice. These outcomes are likely influenced by increased familiarity with digital tools, structured exposure to simulated scenarios, and enhanced communication awareness during training (73–75). However, confidence does not necessarily translate into sustained competence or improved patient outcomes, highlighting the need for more robust evaluation frameworks that assess behavioral change and clinical effectiveness over time.

A qualitative study exploring expert perspectives identified essential competencies for effective virtual care, including workflow integration, adaptation of communication strategies, and appropriate triage decisions regarding in-person vs. remote care (8). Furthermore, systematic review evidence suggests that virtual consultations can achieve outcomes comparable to those of face-to-face care in certain contexts, reduce waiting times, and improve efficiency, although evidence on safety and equity remains limited (72).

Evidence syntheses also indicate that digital health adoption is influenced by factors such as infrastructure limitations, workload pressures, and psychological resistance, whereas structured training and perceived usefulness function as key enablers (73). These findings reinforce the importance of educational interventions within broader digital transformation strategies.

An important consideration emerging from this review is the distinction between synchronous video-based consultations and asynchronous text-based communication. These modalities differ substantially in their communication dynamics, clinical assessment capabilities, and required competencies. Video-based consultations allow for real-time interaction and limited visual assessment, whereas text-based communication relies heavily on written clarity, delayed responses, and structured information exchange. Despite these differences, the included studies often treated telehealth training as a unified concept, limiting the ability to draw modality-specific conclusions. This represents an important gap in the literature and highlights the need for more differentiated training models and evaluation approaches. Overall, these findings suggest that the effectiveness of telehealth training is influenced not only by content but also by delivery modality, duration, and the extent to which training is integrated into clinical practice.

### Conceptual framework for telehealth training

To further integrate the findings, a conceptual framework for telehealth training effectiveness can be proposed based on the included evidence. This framework suggests that training effectiveness is influenced by three interrelated domains: (1) training design characteristics, including delivery modality (e.g., online, face-to-face, simulation-based), duration, and instructional strategies; (2) learner-related factors, such as prior experience with digital technologies, attitudes toward telehealth, and baseline competencies; and (3) contextual and organizational factors, including technological infrastructure, workflow integration, and institutional support. These domains interact to shape intermediate outcomes such as confidence, perceived competence, and readiness, which may subsequently influence clinical practice and, potentially, patient and organizational outcomes. However, the current evidence base remains limited in its ability to demonstrate these downstream effects (72–75). Taken together, these findings highlight the need to move beyond descriptive reporting toward integrated models that explain how and why telehealth training interventions produce their observed effects.

**Figure 2:**
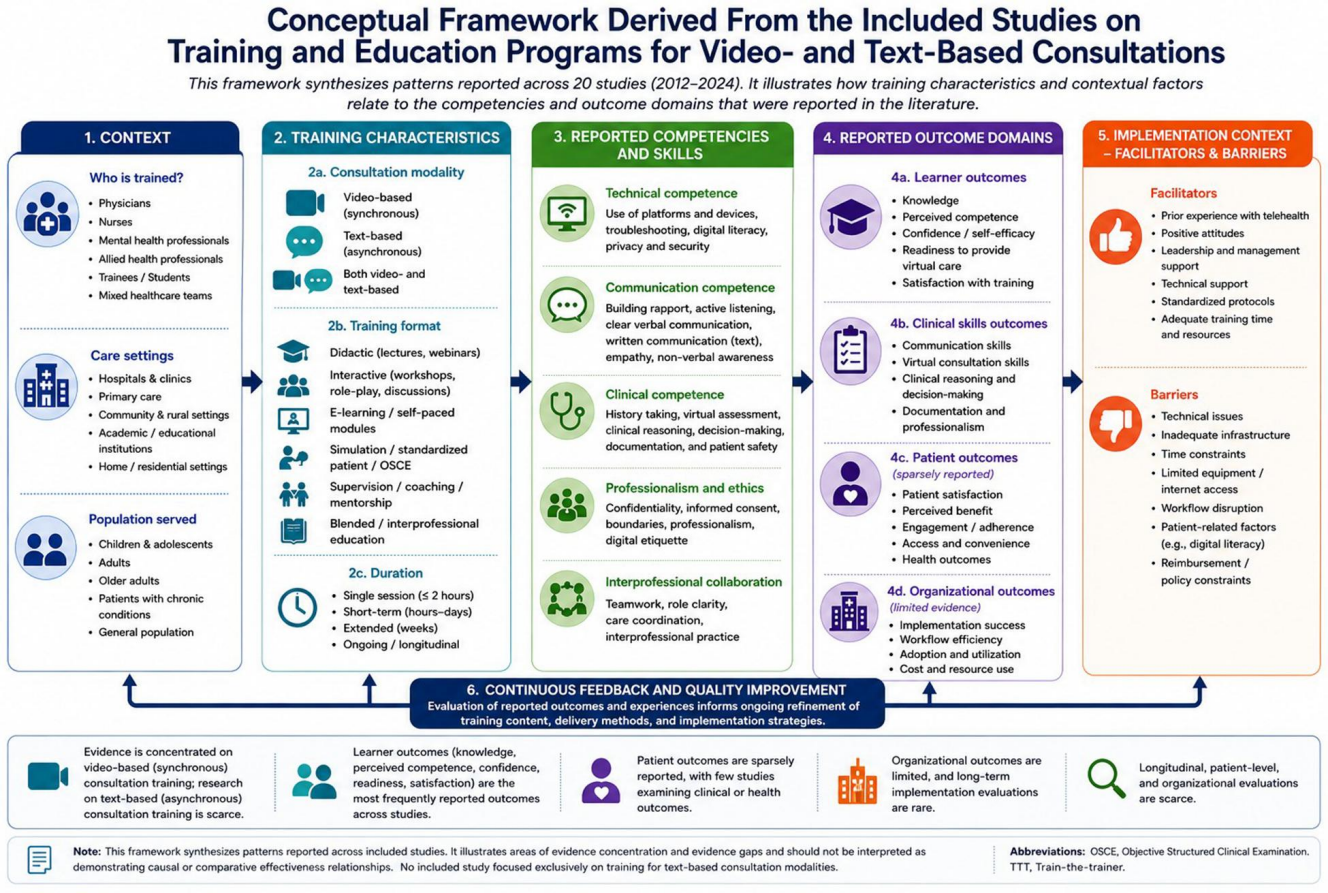
Conceptual framework derived from the included studies on training and education programs for healthcare professionals conducting video- and text-based consultations.

Figure 2 presents a conceptual framework derived from the included studies and summarizes the principal patterns identified across training approaches, competency development, implementation factors, and reported outcomes. This framework synthesizes patterns reported across included studies. It illustrates areas of evidence concentration and evidence gaps and should not be interpreted as demonstrating causal or comparative effectiveness relationships.

This figure presents a conceptual synthesis of the evidence identified in the included studies. The framework illustrates how training characteristics (e.g., delivery format, duration, and consultation modality) are associated with the development of telehealth-related competencies and reported outcomes across learner, patient, and organizational domains. The framework also highlights commonly reported facilitators and barriers influencing implementation. As this review was conducted using a scoping review methodology, the figure represents patterns and relationships reported in the literature rather than causal or effectiveness-based associations.

#### Evaluation methods and evidence gaps

Most included studies assessed outcomes using self-reported measures of competence and confidence or educator-led evaluations conducted in simulated environments (51, 53, 54, 57, 59). Although these approaches provide insight into immediate learning outcomes, they offer limited evidence regarding long-term skill retention or impact in real-world clinical settings (73, 75).

While pre- and post-intervention assessments reduce recall bias (51, 53, 54, 57, 59, 67, 68), the predominance of short-term evaluation designs leaves a substantial gap in understanding sustained behavioral change and patient-level outcomes. Data on the broader organizational impact of telehealth training programs were also limited (72, 73, 75).

Importantly, the limited evidence on patient and organizational outcomes restricts understanding of whether improvements in confidence and perceived competence translate into meaningful changes in healthcare delivery. Few studies evaluated patient satisfaction, care quality, clinical outcomes, workflow efficiency, service performance, or long-term implementation success. As telehealth becomes increasingly embedded in routine care, future research should move beyond learner-focused evaluations and examine how training influences patient experiences, organizational performance, and healthcare quality outcomes over time (72, 73, 75).

For example, one interventional study demonstrated that structured telehealth communication and etiquette training significantly improved provider readiness and knowledge, with potential implications for patient satisfaction and quality of care (78). Nevertheless, robust longitudinal and system-level outcome data remain scarce. Further research is needed to evaluate real-world implementation, patient experiences in routine clinical settings, and organizational performance metrics following telehealth training interventions (72).

The heterogeneity of study designs and the predominance of descriptive and self-reported outcomes further limit the internal validity of the evidence base. The absence of standardized outcome measures and the limited use of robust experimental designs highlight the need for more rigorous evaluation approaches in future research (73–75). The inclusion of heterogeneous study designs without consistent evaluation frameworks further limits the interpretative strength of the findings, underscoring the importance of considering study design and methodological robustness when interpreting the evidence base.

The limited evaluation of patient-level and organizational outcomes represents a significant gap in the current evidence base. While most studies focused on immediate, self-reported outcomes such as confidence and perceived competence, the absence of robust data on patient outcomes (e.g., quality of care, patient satisfaction, clinical effectiveness) and organizational impacts (e.g., workflow efficiency, service utilization, and cost-effectiveness) restricts the ability to assess the real-world value of telehealth training programs. This gap limits the translation of training outcomes into meaningful healthcare improvements and highlights the need for more comprehensive evaluation frameworks (72–75).

A broader observation arising from this review is that telehealth competencies remain inconsistently defined across studies. Although many training programs aimed to improve confidence, communication, and readiness to provide virtual care, there was considerable variation in how these competencies were conceptualized, taught, and evaluated. The absence of consistently defined competency frameworks may hinder the development, implementation, and assessment of training programs, making it difficult to compare outcomes across studies and establish best practices. Future research should focus on developing and validating standardized competency frameworks for video- and text-based consultations to support curriculum design, professional development, and evaluation across healthcare settings.

### Limitations

Several limitations should be acknowledged. As a scoping review, this study did not formally appraise the methodological quality of included studies, and most reported short-term, self-reported outcomes rather than long-term clinical or organizational effects (51–59, 67, 68). Although grey literature sources were searched, no unpublished studies met the inclusion criteria, and only English-language publications were included. Additionally, given the rapidly evolving nature of digital health, emerging training models may not have been captured despite the search being updated through December 24, 2024. While the review was conducted using established methodological frameworks and informed by prior experience in related evidence syntheses (79–81), these limitations should be considered when interpreting the findings. Additionally, the available evidence was overwhelmingly focused on video-based consultations, with no included study specifically evaluating training for text-based consultation modalities.

### Implications for practice, policy, and future research

Building on these findings and identified evidence gaps, several implications for practice, policy, and future research can be considered. For instance, digital transformation in healthcare requires more than technical proficiency; it demands ongoing professional development aligned with evolving clinical, ethical, and organizational requirements (24, 26–28). Training programs must therefore address not only platform navigation but also confidentiality, data governance, interdisciplinary communication, and equity considerations.

Given the sustained integration of video- and text-based consultations into routine practice (6, 39, 72), telehealth education should be embedded within formal curricula and continuing professional development frameworks (18, 38, 74). Sustainable digital health transformation depends on structured competency development, organizational support, and systematic evaluation of educational effectiveness (30–34, 73).

This review identified important evidence gaps, particularly regarding patient outcomes, organizational impact, and long-term effects of telehealth training. Although learner outcomes were frequently reported, relatively little evidence examined whether training translated into improvements in patient experiences, healthcare quality, workflow integration, or organizational performance. Future studies should examine both short-term and sustained effects of digital training programs on patients, healthcare professionals, and healthcare organizations. Broader gaps in telemedicine research agendas, including the influence of training on adoption and implementation outcomes, also warrant attention (82).

Further investigation is needed to assess the impact of telehealth training on patient-level outcomes, including early identification of healthcare needs, reduced waiting times, decreased hospital admissions, improved adherence, and increased patient satisfaction, and to clarify the mechanisms by which training contributes to these outcomes (83). Research should also explore patients' experiences of telehealth-enabled care, including their perceptions of digital comfort and usability. Future research should also explicitly differentiate between video-based and text-based modalities when designing and evaluating telehealth training interventions to ensure that modality-specific competencies are adequately addressed.

Given the heterogeneity of study designs, interventions, and outcome measures, the current evidence base does not permit conclusions regarding the comparative effectiveness of different telehealth training approaches. However, recent evidence suggests that telehealth education may influence the effectiveness of healthcare delivery, underscoring the need to link educational initiatives to measurable outcomes (84). Therefore, future research should incorporate more comprehensive and standardized outcome measures to capture the broader impact of telehealth training. At the patient level, relevant indicators may include patient satisfaction, accessibility of care, clinical outcomes, and patient engagement (38, 85, 86). At the organizational level, indicators such as workflow efficiency, service delivery outcomes, cost-effectiveness, and integration of telehealth into routine practice should be considered. Incorporating these measures would enable a more complete assessment of the effectiveness and sustainability of telehealth training interventions (72–75).

## Conclusion

This scoping review mapped the available evidence on training and education programs designed to prepare healthcare professionals for video- and text-based consultations. To our knowledge, this is the first scoping review to comprehensively synthesize training approaches across both synchronous (video-based) and asynchronous (text-based) modalities, integrating evidence from diverse professional groups, educational settings, and healthcare contexts. By consolidating fragmented literature across disciplines and delivery formats, this review provides a structured overview of current telehealth education practices and identifies key gaps in evaluation, standardization, and long-term impact assessment. The review also identified a substantial evidence gap relating to training for text-based consultation modalities, despite their growing use in contemporary healthcare delivery.

The findings demonstrate considerable variability in instructional design, delivery formats, duration, and outcome evaluation strategies. Across diverse settings, most training interventions were associated with improvements in participants' confidence, perceived competence, and readiness to deliver virtual care, with online and hybrid formats frequently used to support skill development.

However, the evidence base remains limited by short-term evaluations and a predominance of self-reported outcomes. Challenges related to virtual physical examination, patient education, and technological barriers highlight the need for more comprehensive and practice-oriented training models. As digital consultations continue to be integrated into routine healthcare delivery, telehealth education should be embedded within structured curricula and continuing professional development frameworks. Specifically, curricula should incorporate modality-specific competencies (e.g., communication skills for video-based consultations and structured documentation for text-based interactions), simulation-based training (e.g., teleOSCEs), and supervised clinical practice to enhance skill transfer. At the policy level, healthcare organizations should support standardized telehealth competency frameworks, ongoing professional development programs, and integration of telehealth training into routine clinical workflows. In clinical settings, implementation strategies should include structured guidelines, technical support systems, and mechanisms for continuous performance evaluation.

A notable finding of this review was the imbalance in outcome evaluation across the literature. While learner confidence, competence, and readiness are commonly assessed, evidence regarding patient outcomes, organizational performance, and long-term implementation remains limited. Addressing these gaps should be a priority for future research on telehealth education. Future programs may consider incorporating modality-specific competencies, structured experiential learning approaches, and integration into clinical workflows, as these components were commonly reported across the included studies.
