# Supplementary material for: The impact of training and education programs for healthcare professionals on video- and text-based consultations in ensuring healthcare quality: a scoping review
Source: Front Digit Health. 2026 Jul 15;8:1861018. doi: 10.3389/fdgth.2026.1861018 (PMC13415366; doi:10.3389/fdgth.2026.1861018)
Supplement: Supplementary file 1 [file Supplementaryfile1.docx]

## Multimedia Appendix 2: Detailed Search Strategies for All Databases (PRISMA-S Reporting)

## Overview

The search strategy was developed in consultation with an academic librarian and structured in accordance with the PRISMA-S (Preferred Reporting Items for Systematic Reviews and Meta-Analyses Literature Search Extension) guidelines to ensure transparency, reproducibility, and completeness of reporting. The search strategy was iteratively refined through multiple rounds of testing and adjustment to ensure comprehensive coverage of relevant terminology across databases.

All databases were searched from **January 1, 2003, to December 24, 2024**. The year 2003 was selected as the uniform starting point to ensure methodological consistency across databases and to capture literature reflecting modern internet-enabled telehealth practices. Although some databases indexed literature prior to 2003 (e.g., CINAHL from 1990 and PubMed from 1992), APA PsycINFO indexed relevant records from 2003 onward.

Controlled vocabulary (e.g., MeSH terms in PubMed and subject headings in CINAHL and PsycINFO) and free-text keywords were combined using Boolean operators (AND, OR). Truncation symbols (*) and field modifiers (e.g., title and abstract fields) were applied as appropriate for each database.

## 1. PubMed (MEDLINE via PubMed)

**Platform:** National Library of Medicine
**Date searched:** December 24, 2024
**Date range:** January 1, 2003 – December 24, 2024
**Results retrieved:** 1751

**Search Strategy:**

*("Telemedicine" OR "Remote Consultation" OR "Videoconferencing" OR telemedicine OR telehealth OR "video consultation*" OR "video visit*" OR "virtual consultation*" OR "virtual visit*" OR "text-based communication" OR "secure messaging" OR email OR chat OR "patient portal*")*

*AND*

*("Education" OR "Inservice Training" OR "Education, Continuing" OR training OR education OR curriculum OR workshop* OR competenc* OR skill* OR "professional development")*

*AND*

*("Health Personnel" OR clinician* OR physician* OR nurse* OR "healthcare professional*" OR "health care provider*")*

**Limits applied:**

- English language
- Publication date from 2003 onward

## 2. CINAHL (via EBSCOhost)

**Platform:** EBSCOhost
**Date searched:** December 24, 2024
**Date range:** January 1, 2003 – December 24, 2024
**Results retrieved:** 1018

**Search Strategy:**

*("Telemedicine" OR "Videoconferencing" OR telemedicine OR telehealth OR
"video consultation*" OR "virtual visit*" OR "text-based communication" OR
"secure messaging" OR email OR chat OR "patient portal*")
AND
("Education" OR "Inservice Training" OR training OR education OR
curriculum OR workshop* OR competenc* OR skill*)
AND
("Health Personnel" OR clinician* OR physician* OR nurse* OR
"healthcare professional*" OR "health care provider*")*

**Limits applied:**

- English language
- Publication year ≥ 2003

## 3. PsycINFO (via EBSCOhost)

**Platform:** EBSCOhost
**Date searched:** December 24, 2024
**Date range:** January 1, 2003 – December 24, 2024
**Results retrieved:** 631

**Search Strategy:**

*("Telemedicine" OR "Online Therapy" OR telemedicine OR telehealth OR "video consultation*" OR "virtual visit*" OR "text-based communication" OR "secure messaging" OR email OR chat)

AND

("Professional Education" OR "Inservice Training" OR training OR education OR
curriculum OR workshop* OR competenc* OR skill*)

AND

("Health Personnel" OR clinician* OR physician* OR nurse* OR
"healthcare professional*")*

**Limits applied:**

- English language
- Publication year ≥ 2003

## 4. APA PsychArticles (via EBSCOhost)

**Platform:** EBSCOhost
**Date searched:** December 24, 2024
**Date range:** January 1, 2003 – December 24, 2024
**Results retrieved:** 20

**Search Strategy:**

("Telemedicine" OR telemedicine OR telehealth OR "video consultation*" OR "virtual visit*" OR "text-based communication" OR "secure messaging" OR
email OR chat)
AND
("Education" OR training OR education OR curriculum OR workshop* OR competenc* OR skill*)
AND
("Health Personnel" OR clinician* OR physician* OR nurse* OR "healthcare professional*")

**Limits applied:**

- English language
- Publication year ≥ 2003

## 5. Additional Sources (Grey Literature and Manual Searching)

Additional records were identified through grey literature searches and manual methods.

### OCLC WorldCat

**Date searched:** December 24, 2024
**Date range:** 2003 – December 24, 2024

**Search Strategy:**
("telemedicine" OR "telehealth")

AND

("training" OR "education")

AND

("healthcare professional*" OR clinician* OR nurse* OR physician*)

### ProQuest Dissertations and Theses

**Date searched:** December 24, 2024
**Date range:** 2003 – December 24, 2024
**Search Strategy:**

("telemedicine" OR "telehealth")

AND

("training" OR "education")

AND

("healthcare professional*" OR clinician* OR nurse* OR physician*)

Additional identification methods included:

- Citation tracking of included studies
- Manual screening of reference lists

**Total records identified through additional sources:** 576

**Record Management and Deduplication**

All identified records (n = 3996) were imported into Covidence for screening. Duplicate records were identified and removed automatically by Covidence (n = 1014) and manually (n = 1) prior to title and abstract screening. After deduplication, 2981 records remained for screening.
